# Multiscale characterization and contact performance analysis of machining surfaces

**DOI:** 10.1038/s41598-023-36907-6

**Published:** 2023-06-15

**Authors:** Ling Li, Wang Zhang, Jingjing Wang, Xiaoguang Ruan, Lixia Li, Miaoxia Xie

**Affiliations:** School of Mechanical and Electrical Engineering, Xi’an University of Architecture and Technology, Xi’an, 710055 Shaanxi China

**Keywords:** Engineering, Mechanical engineering

## Abstract

Accurately characterizing the surface topography of parts is crucial to improve the surface measurement accuracy and analyze the surface contact performance. A method is proposed to separate the morphological characteristics of the actual machined surface based on the layer-by-layer error reconstruction method and the signal-to-noise ratio method during the wavelet transform process, so as to evaluate the contact performance of the different joint surfaces. First, the actual machined surface morphological features are separated by using the wavelet transform method, the layer-by-layer error reconstruction method, and the signal-to-noise ratio method. Second, the reconstructed three-dimensional surface contact model is established by the reverse modeling engineering method. Third, the finite element method is used to analyze the impact of processing methods and surface roughness on contact surface parameters. The result demonstrates that the simplified and efficient three-dimensional reconstructed surface is achieved based on the real machining surface in contrast to other existing approaches. The surface roughness has a more significant influence on contact performance. The contact deformation increases with the increase of surface roughness, while the curves of average contact stress, contact stiffness, and contact area have the opposite trend.

## Introduction

The surface of the parts leaves slight and uneven scratches during the mechanical machining process, which are composed of morphological features at different scales when viewed microscopically^1–3^. The asperities with different scales and shapes only participate in contact when the surfaces of two parts are in contact, which significantly affects the contact performance of mating surfaces^4, 5^. Therefore, accurately characterizing the surface topography of parts is crucial to improve the surface measurement accuracy and analyze the surface contact performance.

Due to the limitations of the Fourier transform, the traditional surface filtering technology has problems such as surface information homogenization and boundary distortion^6–8^. Nevertheless, the wavelet transform theory is more effective than Fourier transform in solving many problems. This is because the wavelet transform is the combination of scaling and translation, which can carry out the comprehensive multi-scale analysis of various frequency signals. However, the optimal wavelet basis function and the optimal decomposition level are directly related to the correctness of the reconstructed surface extraction during the wavelet decomposition process. The selections of wavelet basis function and decomposition level are of great significance to the accurate characterization of the surface. Therefore, many scholars have conducted in-depth research on the optimizations of wavelet basis functions and decomposition layers. Fu and Shi et al.^9, 10^ investigated the transmission and phase characteristics of Daubechies, Coiflets, and Biorthogonal wavelet families and then employed the bior6.8 wavelet basis functions to perform multi-scale decomposition and reconstruction of three-dimensional surfaces. Xin et al.^11^ compared the filtering performance of various Daubechies wavelet families on multiple groups of signals and adopted the db6 as the optimal wavelet basis function to decompose input signals. Zou et al.^12^ selected multiple wavelet basis functions to determine the optimal wavelet basis function by trial-and-error method for the multi-scale characterization of the surface. By combining artificial neural network theory, Mezghani et al.^13^ were able to determine the optimal wavelet function for multi-scale characterization of the surface. A method based on the combination of power spectral density was proposed by Sun et al.^3^ to accurately obtain the characteristics of various scales of the surface morphology of parts. An and Liu et al.^14, 15^ determined the maximum decomposition scale by evaluating the size of the surface sampling matrix and then employed wavelet transform theory to effectively characterize the mechanical surface at multiple scales. According to the energy conservation method and the characteristics of exponential change in the wavelet decomposition process, Liu et al.^16^ fitted the high-frequency energy under the decomposition coefficient of each layer exponentially and determined the number of decomposition layers corresponding to the energy mutation point as the maximum decomposition layer. Based on the wavelet transform theory, Yuan et al.^17^ used energy entropy ratio and energy methods to characterize the three-dimensional morphology of rock surfaces at multiple scales.

The above research determines the optimal wavelet basis function and decomposition level by analyzing the characteristics of different wavelet basis functions and combining the research results of scholars. However, the above optimization methods are cumbersome, and the experience summary method may not be accurate for different surfaces. Therefore, further research is needed in the optimizations of the wavelet basis function and the decomposition layer.

This study provides a more effective method for multi-scale characterization of surface morphology of mechanical parts based on wavelet transform theory and provides necessary theoretical data support for analyzing the contact performance of joint surfaces and improving the surface quality of mechanical parts.

The remaining sections of this study are proposed in the following manner. The wavelet transform theory, layer-by-layer reconstruction error method, and signal-to-noise ratio method are introduced respectively and the multi-scale characterization of the measured surface is carried out in Sect. “Decompose the surface topography parameter”. Section “Reconstruct the contact surface model” verifies the effectiveness of this research method and establishes a three-dimensional surface finite element model. The finite element method is used to analyze and discuss the contact performance of different mechanical surfaces in Sect. “Analysis of contact surface performance”. Lastly, the conclusions are shown in Section “Conclusions”.

## Decompose the surface topography parameter

### Wavelet decomposition model

The surface morphology is constructed by signals of various frequencies. The mathematical model of wavelet decomposition of part surface can be expressed as^16, 18^.

1 $$f(x,y) = s(x,y) + h(x,y),$$

where *f*(*x*,*y*) is the original surface morphology, *h*(*x*,*y*) is the high-frequency component, and *s*(*x*,*y*) is the low-frequency component. The two-dimensional wavelet is applied to decompose the original surface topography *f*(*x*,*y*) of parts. The three-dimensional surface topography *f*(*x*,*y*) is a space function on *L*^2^(*R*), so the expansion of *f*(*x*,*y*) on orthogonal wavelet bases can be expressed as^19^.

2 $$f(x,y) = f_{j + 1} = f_{j} + g_{j} = \sum\limits_{k \in Z} {c_{j,k} \Phi_{j,k} (x)} + \sum\limits_{i = 1}^{j} {\sum\limits_{k \in Z} {d_{{_{i,k} }} \varphi_{i,k} (x)} } ,$$

where *c*_*j,k*_ is the coefficient corresponding to the scale space, *d*_*j,k*_ is the coefficient corresponding to the wavelet space, *Φ*_*j,k*_(*x*) is the two-dimensional wavelet scale function, and *φ*_*j,k*_(*x*) is the two-dimensional wavelet function. The definition of the two-dimensional wavelet function is as follows^20^.

3 $$\Phi (x,y) = \Phi (x)\Phi (y).$$

The two-dimensional wavelet has three wavelet functions in the horizontal, vertical, and diagonal directions, which are defined as^20^.

4 $$\begin{gathered} \varphi^{H} (x,y) = \Phi (x)\varphi (y) \hfill \\ \varphi^{V} (x,y) = \Phi (y)\varphi (x) \hfill \\ \varphi^{D} (x,y) = \varphi (x)\varphi (y), \hfill \\ \end{gathered}$$

where* f*_*j*_ is obtained by Eq. (2) and the next layer of decomposition is carried out, so *f*_*i*_ = *f*_*i-*1_ + *g*_*i-*1_. After multi-scale decomposition of surface topography *f*(*x*,*y*) layer-by-layer, the algorithm formula of wavelet extraction can be written as^21^.

5 $$f(x,y) = f_{j + 1} = f_{s} + g_{j} + g_{j - 1} + ... + g_{s} .$$

Because *h*(*x*,*y*) belongs to the high-frequency component and *s*(*x*,*y*) belongs to the low-frequency component, which can be acquired by Eq. (5).

6 $$h(x,y) = g_{j} + g_{{j{ - 1}}} + ... + g_{s} ,$$

7 $$s(x,y) = f_{s} .$$

In order to construct the wavelet decomposition model of surface topography better, it is necessary to select the optimal wavelet basis function and the optimal decomposition level.

### The multi-scale separation method of machining surfaces

The multi-scale separation method of machining surfaces is proposed to determine the optimal wavelet basis function and decomposition level in this section. Firstly, the approach of layer-by-layer reconstruction error is adopted to determine the optimal wavelet basis function. Secondly, the method of signal-to-noise ratio is used to determine the optimal decomposition layer.

1. The method of layer-by-layer reconstruction error is used to determine the optimal wavelet basis function. The layer-by-layer reconstruction error method is defined as^22^.
  8 $$E(i) = \max \left( {\left| {X_{0} - X(i)} \right|} \right),$$
  9 $$k_{j} = \frac{{E(i) - b_{j} }}{i},$$
  10 $$E_{m} = \min (k_{j} ),$$

where *E*(*i*) is the reconstruction error of the decomposition of the wavelet of the *i* layer, *X*_*0*_ is the three-dimensional shape of the original surface, *X*_*i*_ is the reconstruction three-dimensional shape after decomposing the *i* layer, *k*_*j*_ is the slope of the straight line after the reconstruction error of *N* layers is fitted by the least square method, *b*_*j*_ is a straight line intercept with the minimum daily multiplication method, *E*_*m*_ is the minimum value of the fit line slope, *m* is the wavelet basis function corresponding to the minimum slope of the fitted straight line (that is, the optimal wavelet basis function), *j* is a various wavelet basis function, *i* takes 1 ~ *N*, *N* is the maximum number of decomposition layer.

Combining Eqs. (9) and (10), the slope of the straight line obtained by diverse wavelet basis functions is compared and analyzed. The overall reconstruction error of the wavelet basis function decreases with the decrease of the slope of the fitted line. Therefore, the wavelet basis function with the smallest slope of the fitted line is selected as the optimal wavelet basis function.

(2) The optimal number of decomposition layer is discussed by the signal-to-noise ratio method. The signal-to-noise ratio is the power ratio of the original three-dimensional surface topography to the layer-by-layer decomposed high-frequency components of the surface, defined as follows^23^
  11 $$SNR(i) = 10\lg \frac{{P_{0} }}{{P_{i} }},$$
  12 $$S_{n} = \max (SNR(i)),$$

where *SNR*(*i*) is the signal-to-noise ratio value, *P*_*0*_ is the original appearance power, *P*_*i*_ is the power of high-frequency components per layer, *S*_*n*_ is the maximum value of signal-to-noise ratio, *n* is the number of small wave analysis layers corresponding to the maximum signal-to-noise ratio (that is, the optimal decomposition layer), *j* is a different small wave basis function selected, *i* takes 1 ~ *N*, *n* is the maximum decomposition layer. The discrete summation method should be used in the calculation of power because the measured part surface data is three-dimensional discrete. The original morphology and high-frequency component power calculation are defined as follows^24^.

13 $$P = \frac{{\sum\nolimits_{{n{ = 1}}}^{N} {f(x_{n} ,y_{n} )^{2} } }}{N},$$

where *P* is the power value, *n* is the number of three-dimensional points on the surface, and *f*(*x*_*n*_,*y*_*n*_) is the value of three-dimensional points.

The decomposition layer corresponding to the peak signal-to-noise ratio is selected as the optimal decomposition layer according to Eqs. (11) and (12). Because the high frequency component of the surface decreases with the increase of signal-to-noise ratio. The surface high-frequency component is the smallest when the signal-to-noise ratio is the largest. Therefore, the number of decomposition layer corresponding to the maximum signal-to-noise ratio value is the optimal number of decomposition layer.

### Experimental analysis

**Figure 1:**
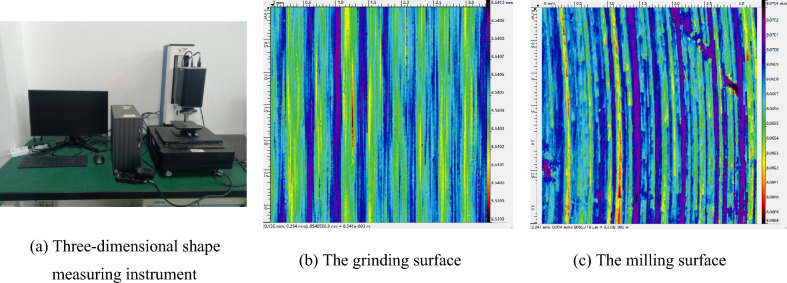
Measured surfaces topography.

The samples used in this study are machined by the actual manufacturers with processing qualifications in Xi’an, Shaanxi Province, China. The grinding and milling surfaces of 45 steel materials are measured using a white light interference profilometer produced by the RTEC company in San Jose, USA. The white light interference profilometer has a lateral resolution of 0.04 μm and a field of view of 3.3 × 3.3 mm. The measured surface topography data is obtained by point-by-point scanning during the measurement process. The three-dimensional topographies of the grinding and milling surface are obtained, as shown in Fig. 1.

The wavelets are frequently used include Daubechies (db*N*) wavelet system, Biorthogonal (biorNr.Nd) wavelet system, Coiflet (coif*N*) wavelet system, Symlets (sym*N*) wavelet system and Morlet(morl) wavelet system, etc. Three common wavelet systems, including db*N* (3 ~ 10), sym*N* (2 ~ 8) and coif*N* (1 ~ 4), are selected to determine the optimal wavelet basis function^17^. Based on the optimization method of the wavelet basis function proposed in 2.2, the reconstruction error and the slope of the fitted line are respectively calculated corresponding to diverse wavelet basis functions by Eqs. (8) and (9), and then the optimal wavelet basis function is determined by Eq. (10).

**Figure 2:**
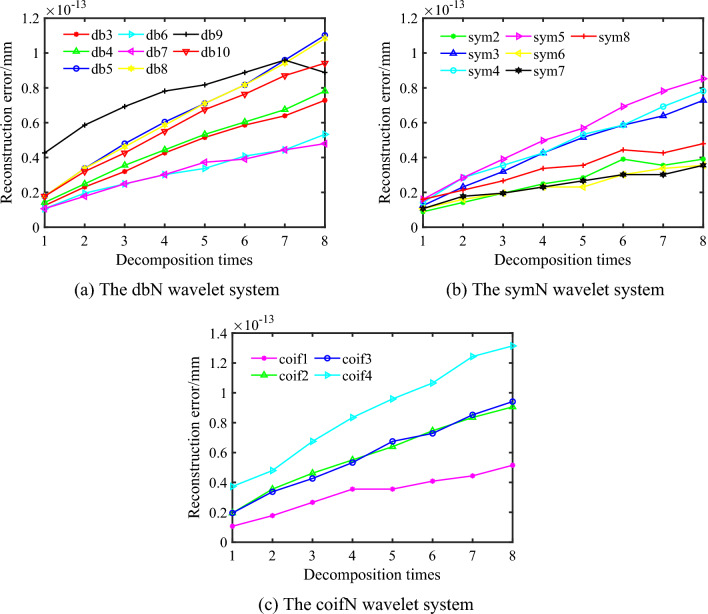
The relationship between layer-by-layer reconstruction error and decomposition times of grinding surface.

**Figure 3:**
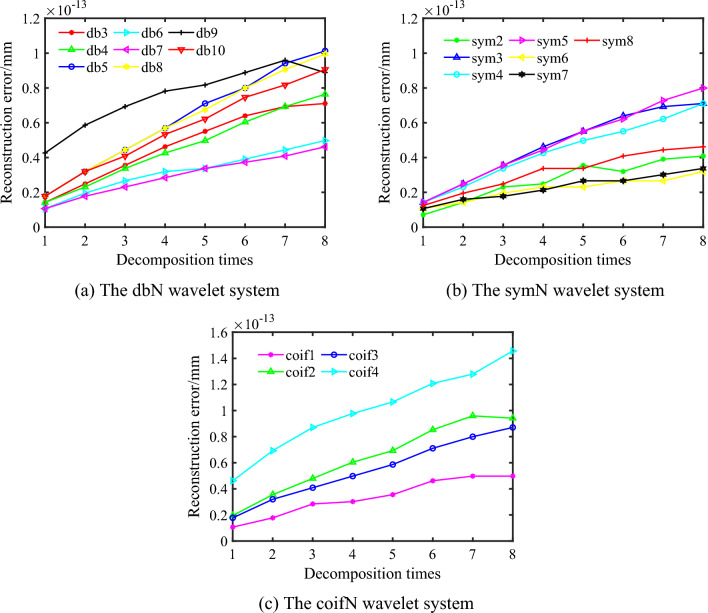
The relationship between layer-by-layer reconstruction error and decomposition times of milling surface.

For the grinding surface, the reconstruction error corresponding to the wavelet basis function of the sym7 is small overall, and the minimum slope of the fitting line is 3.236e–15 according to Eq. (9) as shown in Fig. 2. Consequently, the sym7 wavelet basis function is determined as the optimal wavelet basis function for the grinding surface. Likewise, for the milling surface, the reconstruction error corresponding to the sym6 wavelet basis function is small overall, and the slope of the fitting line is 2.77e–15 according to Eq. (9) as shown in Fig. 3. Accordingly, the sym6 wavelet basis function is determined as the optimal wavelet basis function for the milling surface.

**Figure 4:**
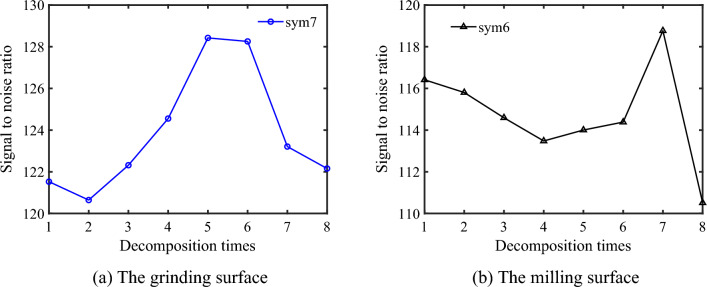
Schematic diagram of signal-to-noise ratio and decomposition times.

Based on the optimization method of the decomposition layer proposed in 2.2, the signal-to-noise ratio of each layer is calculated by Eqs. (11) and (13), and the optimal decomposition layer is determined by Eq. (12). As shown in Fig. 4, the signal-to-noise ratio reaches a peak with the increase of the number of wavelet decomposition layer. This is because the resolution of the low-frequency component gradually improves in the process of decomposition. However, the resolution of the high-frequency component gradually decreases. Accordingly, the resolution of the high-frequency component of the surface is the smallest when the signal-to-noise ratio reaches the peak.

As for the grinding surface, it can be seen from Fig. 4a that the maximum signal-to-noise ratio corresponding to the sym7 wavelet basis function is 129.038, and the corresponding number of decomposition layers is 5. Consequently, the optimal number of decomposition layers on the grinding surface is determined to be 5. Similarly, for the milling surface, the maximum signal-to-noise ratio corresponding to the wavelet basis function of sym6 is 118.766, and the corresponding number of decomposition layer is 7 as shown in Fig. 4b. Therefore, the optimal number of decomposition layers on the grinding surface is determined to be 7.

**Figure 5:**
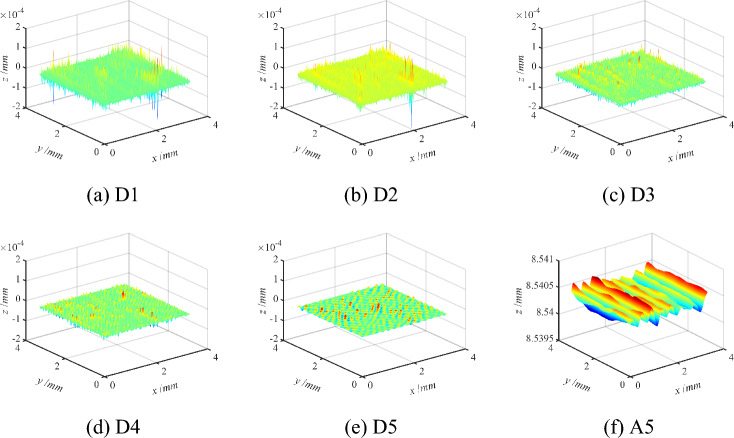
Schematic diagram of multi-scale decomposition of the grinding three-dimensional surface.

**Figure 6:**
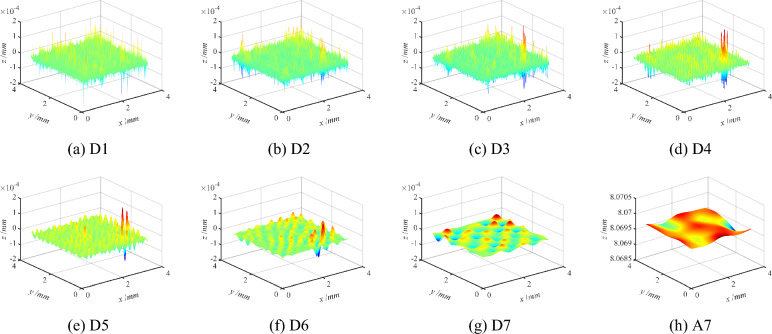
Schematic diagram of multi-scale decomposition of the milling three-dimensional surface.

In summary, the measured surface is characterized at multiple scales after determining the optimal wavelet basis function and the optimal number of decomposition layer. The sym7 wavelet basis function is used to decompose the grinding surface topography into the five layers, and the grinding surface topography characteristics of different scales are obtained as shown in Fig. 5. Similarly, the sym6 wavelet basis function is used to decompose the milling surface topography into seven layers, and then the milling surface topography characteristics of different scales can be obtained as illustrated in Fig. 6.

## Reconstruct the contact surface model

### Reconstruction of surface model validation

**Figure 7:**
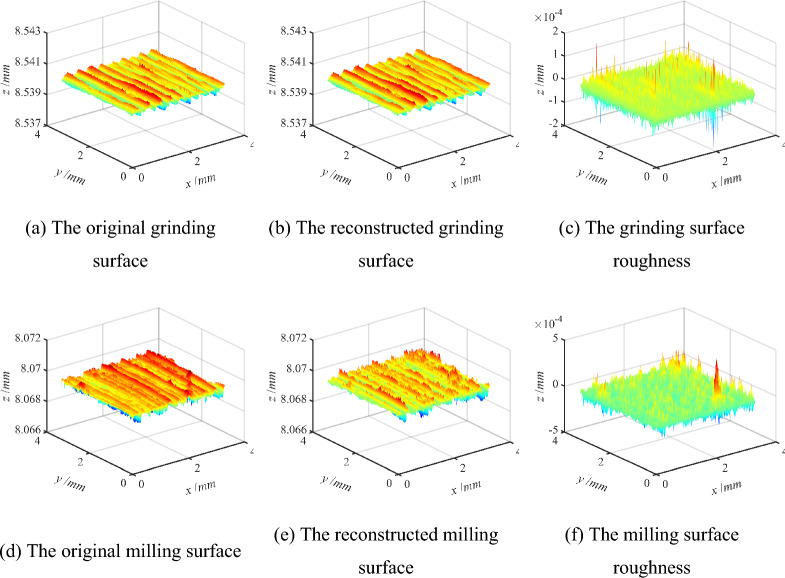
The reconstructed surface and surface roughness.

The different three-dimensional surface topography is decomposed into multiple frequency components according to the analysis of the previous section. The high-frequency component of the measured three-dimensional surface is deleted in order to improve the efficiency and accuracy of the calculation. Therefore, the more realistic reconstructed surface topography data is obtained, as illustrated in Fig. 7.

The parameters used to evaluate the three-dimensional surface roughness reflect the overall characteristics of the surface in space. However, too many surface parameters may cause an explosion of parameters, which is meaningless^24^. Therefore, according to the structure of the machined surface features and the ISO25148-2 standard, the representative three-dimensional surface feature parameters are selected to characterize the machined surface^25, 26^. As shown in Table 1, *Sa*, *Sq*, *Ssk*, *Sku*, *Sp*, *Sv*, and *Sz* are height parameters; *Sdq* and *Sdr* are hybrid parameters^27, 28^. The surface parameter evaluation Gwyddion software is used to process the measured surface data, and the roughness parameters of the grinding surface are obtained as shown in Table 2. In order to ensure the accuracy of the measured data, the data of the three regions of the grinding surface are collected for analysis and processing, and the average value of each parameter is used to characterize the grinding surface morphology. Due to the limitation of table size, only the data of the region 1 and the average value of each parameter are added to Table 2.

**Table 1:** Three-dimensional surface roughness parameters.

Parameters	The meaning and function of parameters	The formula for calculating parameters
Arithmetic mean height (*Sa*)	The arithmetic mean of the absolute values of the waviness profile. It characterizes the more widely spaced surface features	$S_{a} = \frac{1}{A}\iint\limits_{A} {\left| {Z(x,y)} \right|dxdy}$
Root mean square height (*Sq*)	The root-mean-square of the surface roughness profile. It provides a measure of the deviation of the surface from a perfect flatness	$S_{q} = \sqrt {\frac{1}{A}\iint\limits_{A} {Z^{2} (x,y)dxdy}}$
Skewness (*Ssk*)	The skewness of the surface roughness data. It reflects the surface inhomogeneity and asymmetry	$S_{sk} = \frac{1}{{S_{q}^{3} }}\left[ {\frac{1}{A}\iint\limits_{A} {Z^{3} (x,y)dxdy}} \right]$
Kurtosis (*Sku*)	The kurtosis of surface height distribution. It can detect whether there is a peak or valley defect on the surface	$S_{ku} = \frac{1}{{S_{q}^{4} }}\left[ {\frac{1}{A}\iint\limits_{A} {Z^{4} (x,y)dxdy}} \right]$
Maximum peak height (*Sp*)	The maximum peak height of the surface	$S_{p} = \mathop {\max }\limits_{A} Z(x,y)$
Maximum pit height (*Sv*)	The maximum valley depth of the surface	$S_{v} = \mathop {\min }\limits_{A} Z(x,y)$
Maximum height (*Sz*)	The maximum surface height	$S_{z} = S_{p} + S_{v}$
Root mean square gradient (*Sdq*)	The root mean square slope of the three-dimensional surface. It can represent the average magnitude of surface local gradient	$S_{dq} = \sqrt {\frac{1}{A}\iint\limits_{A} {\left[ {\left( {\frac{\partial z(x,y)}{{\partial x}}} \right)^{2} + \left( {\frac{\partial z(x,y)}{{\partial y}}} \right)^{2} } \right]dxdy}}$
Developed interfacial area ratio (*Sdr*)	The ratio of surface expansion interface area. It reflects the ratio of the expansion area of the definition area to the area of the definition area	$S_{dr} = \frac{1}{A}\left[ {\iint\limits_{A} {\left( {\sqrt {\left[ {1 + \left( {\frac{\partial z(x,y)}{{\partial x}}} \right)^{2} + \left( {\frac{\partial z(x,y)}{{\partial y}}} \right)^{2} } \right]} - 1} \right)dxdy}} \right]$

**Table 2:** Comparison of surface parameters between the original grind surface and the reconstructed grind surface.

The statistical parameters	Area 1	Mean value	The model of this article	The method of reference^14^	The method of reference^16^	Relative error of this article (%)	Relative error of reference^14^ (%)	Relative error of reference^16^ (%)
*Sa* (um)	0.16289	0.13572	0.13529	0.13530	0.13500	0.31365	0.30778	0.52939
*Sq* (um)	0.19539	0.16493	0.16433	0.16434	0.16410	0.36024	0.36969	0.50059
*Ssk* (–)	− 0.07602	− 0.09164	− 0.08972	− 0.08972	− 0.08477	2.08908	2.45852	7.49015
*Sku* (–)	2.51196	2.26122	2.61332	2.61234	2.60601	0.30154	0.33913	0.57745
*Sp* (mm)	8.54103	8.54104	8.54103	8.54102	8.54103	1.19E-04	1.34E-04	1.19E-04
*Sv* (mm)	8.53979	8.53979	8.53976	8.53980	8.53977	4.46E-04	6.64E-05	3.38E-04
*Sz* (um)	1.23868	1.23868	1.26657	1.2216	1.25741	2.25178	1.38059	1.51181
*Sdq* (–)	72.94016	72.94007	72.94007	72.94007	72.94007	3.09E-09	6.95E-09	6.57E-06
*Sdr* (%)	0.00657	0.005880	0.005755	0.005753	0.005759	2.11054	2.14408	2.05139

In order to verify the accuracy of the reconstructed surface model, it is inevitable to characterize the reconstructed three-dimensional surface information by surface roughness parameters. According to the formula of roughness parameters in Table 1, the roughness parameters of the reconstructed surface are calculated by MATLAB analysis software. By comparing with the methods used in reference^14^ and reference^16^, the correctness and effectiveness of the three-dimensional reconstructed surface obtained are verified in this study. The reference method^14^ is used to select the db9 wavelet basis function and the nine decomposition layers for multi-scale grinding surface characterization. Similarly, the sym5 wavelet basis function and the five decomposition layers are selected using the reference method^16^. The three-dimensional roughness parameters of the reconstructed surface are calculated, and the results are shown in Table 2.

As can be seen from Table 2, according to the method of this study, the relative errors of *Sa*, *Sq*, *Ssk*, *Sku*, *Sp*, *Sv*, *Sz*, *Sdq*, and *Sdr* between the original grind surface and the reconstructed grinding surface are 0.31365%, 0.36024%, 2.08908%, 0.30154%, 1.19E–04%, 4.46E–04%, 2.25178%, 3.09E–09%, and 2.11054%. Except for the relative error values of roughness parameters *Sa* and *Sz*, which are slightly higher than those calculated in reference^14^, the relative errors of other parameters are smaller than those of reference^14^. Furthermore, the relative error of the roughness parameter *Sdr* is slightly greater than that calculated in reference^16^, while the relative errors of other parameters are smaller than those in reference^16^.

In summary, compared with the methods used in reference^14^ and reference^16^, the relative error of three-dimensional surface roughness obtained by the method proposed in this study is smaller, which shows the correctness and effectiveness of this research method. Meanwhile, the simplified and efficient three-dimensional reconstructed surface is achieved based on the real machining surface. Because the milling surface and the grinding surface adopt the same calculation model, it also shows the effectiveness of reconstructing the milling surface. Therefore, a more accurate and effective three-dimensional reconstructed surface model provides necessary theoretical data support for analyzing the contact performance of the joint surface and improving the surface quality of mechanical parts.

### Finite element model construction

The contact analysis of the reconstructed three-dimensional surface model is carried out according to Sect. “Reconstruction of surface model validation”. The average contact pressure and normal deformation of the rough surface contact body are extracted and fitted in the form of the power exponential function^29^.

14 $$\delta = cp^{m} ,$$

where *c* and *m* are the undetermined coefficients affecting the normal deformation of the joint surface respectively, and the local normal contact stiffness per unit area is expressed as^29^

15 $$k = \frac{dp}{{d\delta }} = \frac{1}{cm}p^{1 - m} ,$$

16 $$k = \alpha p^{\beta } ,$$

where *α* = 1/(*cm*) and *β* = 1-*m*.

In order to explore the influence of different surfaces on contact performance, the surface contact parameters are analyzed under different normal displacements. For a matching node *i* on the surface of 45 steel materials with unusual processing methods, the contact pressure at the node is *p*_*i*_, which has the following relationship with the contact state at the node^3, 30^.

$$p_{i} = \left\{ {\begin{array}{*{20}c} 0 \\ {(0,\infty )} \\ {(0,235MPa]} \\ {(235MPa,\infty )} \\ \end{array} } \right. \, \begin{array}{*{20}c} {\text{Non contact state}} \\ {\text{Contact status}} \\ {\text{ Elastic contact state}} \\ {\text{Plastic contact state}} \\ \end{array} .$$

The percentage of the surface contact area is defined as the percentage of the ratio between the real contact area and the nominal contact area. The contact pressure is extracted at each node, and the nodes are divided into contact nodes, elastic contact nodes, and plastic contact nodes according to the contact state. The percentage of the total number of nodes in each contact state to the total number of nodes on the contact surface is calculated. Therefore, the total contact area, the percentage of elastic contact area, and the percentage of plastic contact area to nominal contact area can be obtained.

**Figure 8:**
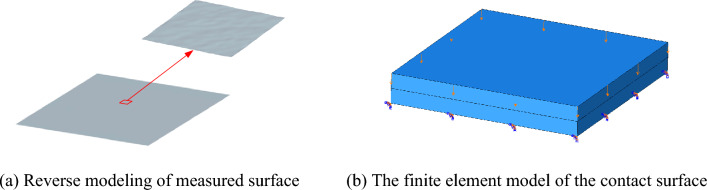
Reverse modeling method of three-dimensional surface.

The three-dimensional reconstructed surface morphology data are extracted by MATLAB software. Then, the extracted three-dimensional coordinate point data is imported into PROE drawing software for solid modeling. Subsequently, the three-dimensional rough surface solid model is imported into ABAQUS finite element analysis software for analysis. The three-dimensional surface finite element model is shown in Fig. 8. The contact surface is set as friction contact, the normal contact as hard contact, the tangential friction coefficient is 0.15, and the lower surface of the lower contact model is completely fixed.

## Analysis of contact surface performance

The finite element method is used to analyze the grinding and milling surface contact models with different roughness from the elastic–plastic contact performance of the contact surface in this section. The effective way to improve surface contact performance is proposed by analyzing the effects of different machining methods and three-dimensional surface roughness on surface performance.

### Average contact stress

Different reconstructed grinding and milling surfaces are taken as research objects in order to analyze the influence of different machining surfaces on contact performance. The measured surfaces mainly include smooth grinding reconstructed surface (*Sa* = 0.6 μm), medium rough grinding reconstructed surface (*Sa* = 0.8 μm), rough grinding reconstructed surface (*Sa* = 1.6 μm), smooth milling reconstructed surface (*Sa* = 1.6 μm), medium rough milling reconstructed surface (*Sa* = 3.2 μm) and rough grinding reconstructed surface (*Sa* = 6.4 μm). The reconstructed surface contact model is established after the multi-scale decomposition of the measured surface. For the convenience of description, the above reconstructed surfaces are recorded as RS-1, RS-2, RS-3, etc.

**Figure 9:**
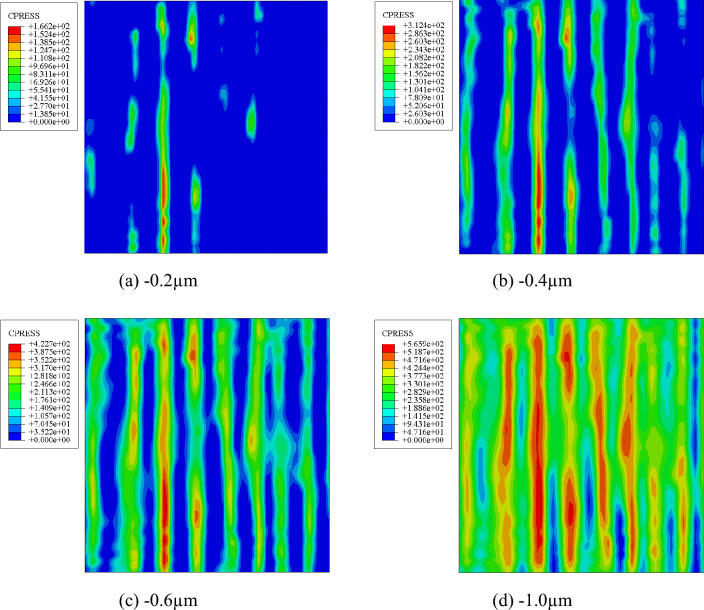
Cloud diagram of the RS-3 pressure changing with the contact displacement.

The various surface contact pressure contours can be obtained by applying different Z-direction displacements during the finite element analysis. This section only gives the contact pressure cloud diagram of the rough grinding reconstructed surface (RS-3) with normal displacements of − 0.2 μm, − 0.4 μm, − 0.6 μm and − 1.0 μm due to limited space, as shown in Fig. 9. The normal displacement and average contact pressure stress curves of different grinding and milling surfaces are obtained by extracting the corresponding values and using the Eq. (14) for fitting.

**Figure 10:**
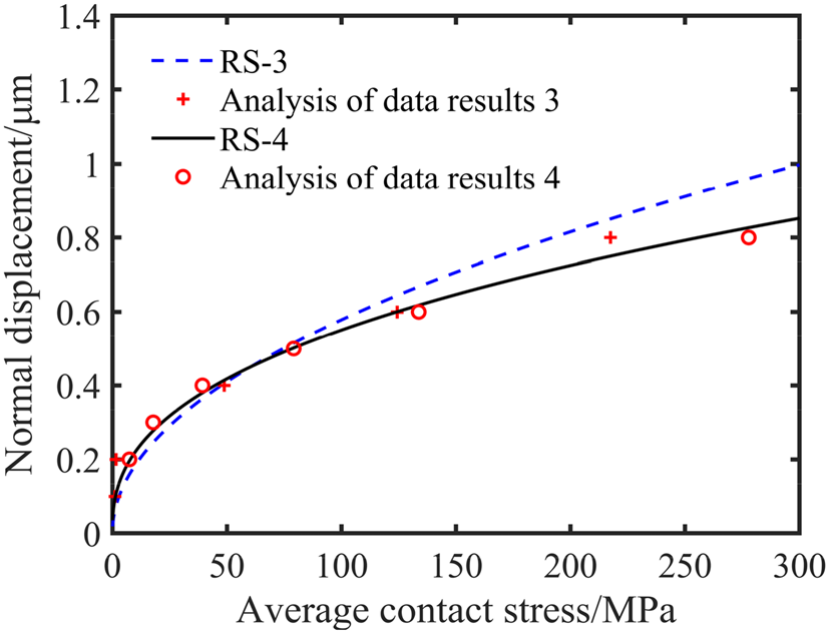
Fitting curves of normal displacement and average contact stress of different machining methods.

The RS-3 and the RS-4 are taken in order to analyze the influence of different processing methods on the average contact pressure as shown in Fig. 10. The average contact pressure of RS-3 is close to that of RS-4 when the normal displacement is small. However, the average contact pressure of RS-3 is slightly larger than that of RS-4 as the normal displacement increases. These indicate that different surface machining methods have a slight effect on the surface average contact pressure in the same case.

**Figure 11:**
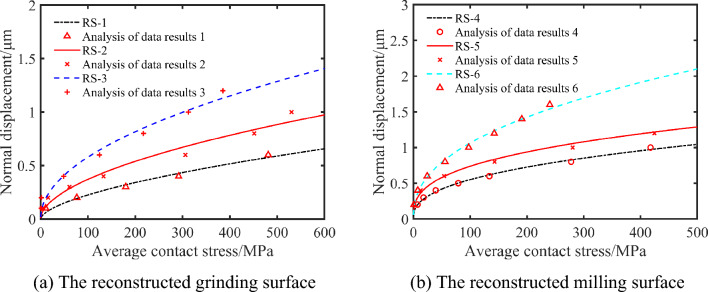
Fitting curves of normal displacement and average contact stress of different three-dimensional surface roughness.

The reconstructed surface with different roughness is used to explore the influence of surface roughness on the average contact pressure. The average contact pressure decreases with the increase of surface roughness in the same case as shown in Fig. 11. This is because the number of contact asperities decreases per unit area with the increase of three-dimensional surface roughness, which leads to the decrease of surface pressure under the same normal displacement.

### Contact stiffness analysis

By analyzing the power exponential function fitting curve of average contact pressure and normal displacement of different surfaces, the values of *α* and *β* required are calculated by Eq. (15), and then the relationship between contact stiffness *k* per unit area and nominal contact pressure *p* is obtained by Eq. (16).

The RS-3 and the RS-4 are calculated to analyze the impact of different machining methods on surface contact stiffness. For the rough grinding reconstructed surface (RS-3), the local normal contact stiffness per unit area can be expressed as

17 $$k_{3} = {34}{\text{.2497}}p_{3}^{0.5037} .$$

Likewise, for the smooth milling reconstructed surface (RS-4), the local normal contact stiffness per unit area can be expressed as

18 $$k_{4} = {34}{\text{.6058}}p_{4}^{{{0}{\text{.6023}}}} .$$

The contact stiffness of the milling surface is slightly higher than that of the grinding surface under the same contact pressure as indicated by Eqs. (17) and (18). This shows that the different surface processing methods have a slight impact on the surface contact stiffness under the same surface roughness.

**Figure 12:**
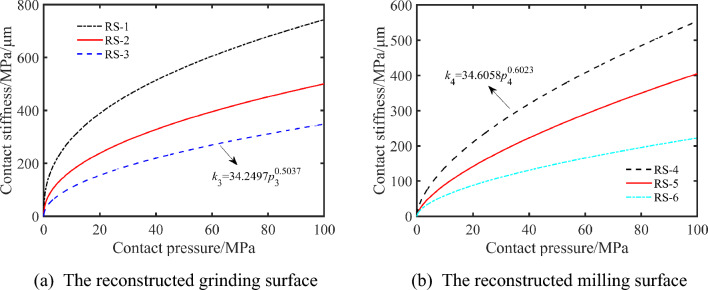
The relationship between normal displacement and contact stiffness of different three-dimensional surface roughness.

The reconstructed surfaces with different roughness are used to analyze the influence of surface roughness on the contact surface stiffness. The surface contact stiffness shows a trend of increasing sharply and then leveling off as the surface contact pressure increases, as demonstrated by Fig. 12. The surface contact stiffness decreases with the surface roughness increases when the surface contact pressure is constant. The surface contact stiffness is more obviously affected by the three-dimensional surface roughness when the contact pressure increases. This is because the ability of the surface to resist deformation decreases with the increase of the three-dimensional surface roughness.

### Contact area analysis

**Figure 13:**
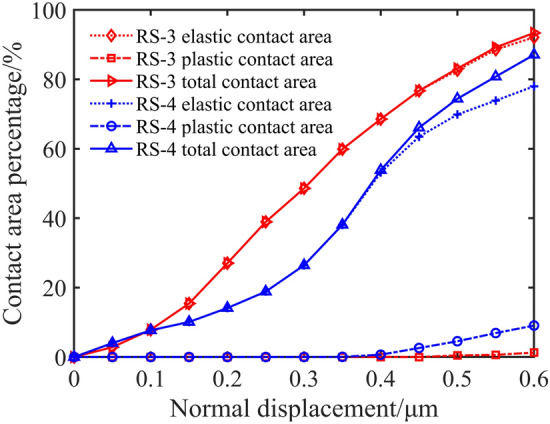
The relationship between normal displacement and contact area of different machining methods.

The percentage of elastic contact area, plastic contact area, and total contact area of different surfaces to nominal contact area can be calculated according to the content of Sect.  “Finite element model construction”. On the one hand, it can be seen from Fig. 13 that the elastic contact area, plastic contact area, and total contact area of RS-3 and RS-4 are close to the same normal displacement. This shows that the machining method has little effect on the surface contact area.

**Figure 14:**
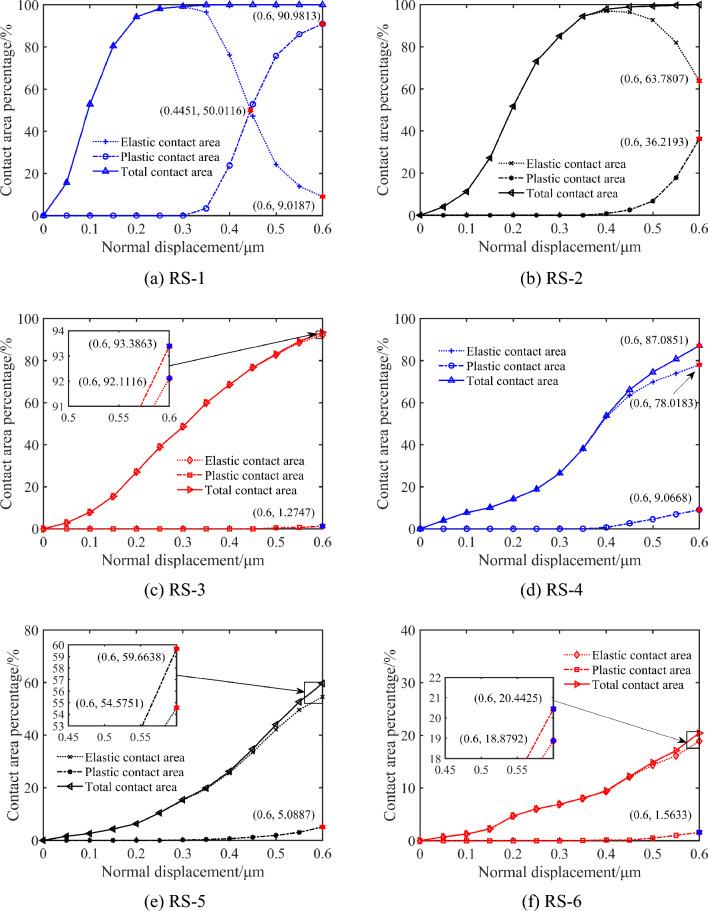
The relationship between normal displacement and contact area of different three-dimensional surface roughness.

On the other hand, as shown in Fig. 14, the surface elastic contact area gradually decreases and the surface plastic contact area rises sharply when the normal displacement reaches a certain level. As shown in Fig. 14a–c, the contact area of RS-1 increases faster than that of RS-2 and RS-3. The RS-1 changes from the elastic contact state to plastic contact state earlier when the normal displacement reaches a certain degree. The plastic contact area of RS-3 is also slowly increasing with the increase of normal displacement. This is due to the different roughness of the reconstructed grinding surface. The plastic contact area of RS-1 with smaller surface roughness changes rapidly, while the plastic contact area of RS-3 with larger surface roughness changes slowly. As shown in Fig. 14d–f, the contact area of RS-4, RS-5 and RS-6 increases slowly with the increase of normal displacement. Compared with the surface with larger roughness, the total contact area of the reconstructed milling surface with smaller roughness accounts for a larger proportion.

The normal displacement and the contact area have certain nonlinearity during the whole loading process, as illustrated in Fig. 14. This is because the percentage of contact area in each contact state gradually increases with the increase of normal displacement. At the same time, the contact state of each node on the surface has undergone a sharp transition from elastic contact state to the plastic contact state when the normal displacement reaches a certain degree. The reconstructed surface with smaller roughness has a larger contact area under the same normal displacement. Compared with different processing methods, the influence of surface roughness on contact performance parameters is more significant. This is because the number of asperities in contact per unit area decreases with the increase of surface roughness, that is, the corresponding contact deformation is obtained with only a small normal displacement, so the contact area is smaller.

## Conclusions

A method is proposed to separate the morphological characteristics of the actual machined surface based on the layer-by-layer error reconstruction method and the signal-to-noise ratio method during the wavelet transform process, so as to evaluate the contact performance of the different joint surfaces. The optimal wavelet basis functions of sym7 and sym6 for the measured grinding surface and milling surface are established by using the layer-by-layer reconstruction error method. Additionally, the optimal decomposition layers of the measured grinding surface and the milling surface are determined to be five layers and seven layers by the signal-to-noise ratio method. Compared with other methods, the relative error of the three-dimensional surface roughness obtained by the method proposed in this study is smaller. Therefore, the simplified and efficient three-dimensional reconstructed surface is achieved based on the real machining surface. By analyzing a three-dimensional reconstructed surface finite element model, it is determined that the surface roughness has a more significant impact on the contact performance parameters compared with the various machining methods. The contact deformation increases with the increase of surface roughness in the same case, while the curves of average contact stress, contact stiffness, and contact area have the opposite trend. The surface contact performance is mainly obtained by applying different normal displacements to the reconstructed surface to obtain the surface contact parameters in this study, without considering the change of the surface contact state during the service process. Therefore, the evolution law of contact performance during service can be analyzed in the future to provide theoretical support for improving the overall performance of mechanical structures.

## Data Availability

The datasets used and/or analysed during the current study available from the corresponding author on reasonable request.
